# Development of a Single-Tube Asymmetric ERA-CRISPR/Cas12a Assay for Rapid Visual Detection of *Enterocytozoon hepatopenaei* in Shrimp

**DOI:** 10.3390/microorganisms14061307

**Published:** 2026-06-11

**Authors:** Ren Liu, Sizhi Sun, Yiqi Cao, Zhenyang Ma, Xin Zhou, Jiaojiao Han, Jun Zhou

**Affiliations:** State Key Laboratory for Quality and Safety of Agro-Products, School of Marine Sciences, Ningbo University, Ningbo 315211, China; 15102721800@163.com (R.L.); sun_sizhi@163.com (S.S.); caoyiqi@nbu.edu.cn (Y.C.); 13771144231@163.com (Z.M.); m19856220879@163.com (X.Z.)

**Keywords:** *Enterocytozoon hepatopenaei*, asymmetric enzymatic recombinase amplification, CRISPR/Cas12a, one-pot detection, *Litopenaeus vannamei*

## Abstract

The microsporidian parasite *Enterocytozoon hepatopenaei* (EHP) is a major pathogen causing severe growth retardation in shrimp, leading to substantial economic losses in global aquaculture. To address the urgent need for accurate, rapid, and field-deployable diagnostic tools for EHP, this study developed a novel one-pot detection platform by integrating asymmetric Enzymatic Recombinase Amplification (aERA) with a PAM-independent CRISPR/Cas12a system (AYERA-Cas12a) based on ssDNA activation. This design circumvents the compatibility challenge between isothermal amplification and CRISPR activity in a single tube by generating single-stranded DNA amplicons that activate Cas12a without requiring a PAM sequence. The assay operates at a constant temperature of 46 °C and completes detection within 15 min. It achieves a sensitivity of 10 copies/μL, equivalent to qPCR, and shows no cross-reactivity with six other prevalent shrimp pathogens. Validation using 56 clinical shrimp (*Litopenaeus vannamei*, *L. vannamei*) samples demonstrated complete agreement with qPCR results. With its simple procedure, isothermal conditions, and clear endpoint fluorescence readout under blue light, the AYERA-Cas12a platform is suitable for point-of-care testing (POCT). This work provides a user-friendly tool for the on-site surveillance and early diagnosis of EHP, offering significant potential for improving disease management in shrimp farming.

## 1. Introduction

The accurate and timely detection of *Enterocytozoon hepatopenaei* (EHP) poses a critical challenge in managing biosecurity within aquaculture systems [1]. As a microsporidian parasite, EHP causes hepatopancreatic microsporidiosis, resulting in severe growth retardation and substantial economic losses estimated to exceed hundreds of millions of dollars annually [2,3]. The importance of controlling this pathogen extends beyond economic impaction; ineffective management can lead to the misuse of antimicrobials; exacerbating issues related to antimicrobial resistance and environmental pollution [4,5,6]. Furthermore, as a potential contaminant, EHP poses a latent risk to overall food safety and ecosystem health [7]. Consequently, developing effective monitoring tools for EHP is crucial not only for animal health but also for mitigating broader environmental and public health risks.

Current diagnostic methodologies for EHP face limitations that hinder their effectiveness for on-site, early-warning applications [8]. A range of molecular detection methods has been developed and implemented in response to the urgent need for sensitive and specific identification of EHP in cultured prawns, a key step toward reducing associated economic losses [9]. Although polymerase chain reaction (PCR) and quantitative PCR (qPCR) demonstrate high sensitivity and specificity, they require sophisticated, centralized laboratory infrastructure, resulting in delays between sampling and result interpretation that can be detrimental for timely interventions [10,11,12]. Immunochromatographic lateral flow assays (LFAs), designed for point-of-care (POC) use, frequently lack the necessary sensitivity to detect low-level, early-stage infections [13,14]. This diagnostic gap underscores the urgent need for technology that merges high analytical sensitivity with the practicality of field-deployable platforms.

To address this need, isothermal nucleic acid amplification techniques, such as Loop-mediated Isothermal Amplification (LAMP), Recombinase Polymerase Amplification (RPA), and the Enzymatic Recombinase Amplification (ERA), employed in this study, provide a robust foundation [15,16]. These methods facilitate rapid target amplification at a constant low temperature, thereby eliminating the need for thermal cyclers [17]. The recent integration of these techniques with CRISPR-Cas systems has dramatically advanced the field of molecular diagnostics [18]. The CRISPR-Cas12a system, a class 2 type V CRISPR-associated nuclease, exhibits non-specific single-stranded DNA (ssDNA) trans-cleavage activity upon specific recognition of its target DNA sequence [19,20]. This collateral cleavage effect can be harnessed to degrade a labeled reporter molecule, thereby converting the specific nucleic acid recognition event into an easily interpretable fluorescent or lateral flow signal [21]. The synergy between isothermal amplification and CRISPR-Cas detection, as evidenced by platforms like RPA/LAMP-CRISPR, has been successfully applied for the detection of various human and animal pathogens, including SARS-CoV-2, African swine fever virus, and *Mycobacterium tuberculosis* [22,23,24]. These applications highlight the technology’s core strengths: exceptional sensitivity, single-base specificity, and compatibility with simple readouts [25,26]. However, limitations persist, often related to the optimization of reaction conditions to prevent non-specific amplification, the stability of reagents in field settings, and the need for streamlined, single-tube formats to minimize cross-contamination and operational complexity [27,28].

A more fundamental limitation, which we term the “fundamental incompatibility”, arises when isothermal amplification and CRISPR-Cas12a detection are combined in a one-pot format. This incompatibility occurs because conventional Cas12a-crRNA complexes recognize double-stranded DNA (dsDNA) targets in a PAM-dependent manner. If the Cas12a system is present during the early stage of amplification, it can bind to and cleave the nascent dsDNA amplicons that contain a PAM motif, thereby destroying the amplification template and severely compromising reaction efficiency [29]. Several elegant strategies have been developed to overcome this problem, including physical separation of amplification and detection (e.g., glycerol barriers [30], sucrose gradients [31], or photocontrolled release [32]), buffer engineering, and the use of engineered Cas variants with relaxed PAM requirements [33]. While effective, these approaches often require additional handling steps, specialized reagents, or extended reaction times. An alternative and potentially more streamlined solution is to change the nature of the activation substrate: Cas12a is known to exhibit robust trans-cleavage activity upon binding to single-stranded DNA (ssDNA) targets without the need for a PAM sequence [34]. Therefore, if an amplification method can produce abundant ssDNA instead of dsDNA, the Cas12a system could be activated without ever cleaving the initial dsDNA templates, thus resolving the one-tube incompatibility at its source.

Herein, this study presents a novel assay that integrates asymmetric enzymatic recombinase amplification with CRISPR/Cas12a (AYERA-Cas12a), utilizing EHP as a representative model to establish a robust and high sensitivity detection platform. The specificity of the AYERA-Cas12a assay was evaluated against a panel of six common shrimp pathogens, showing no cross-reactivity with any of them. Further validation with a broader range of targets and interlaboratory studies would be needed to fully establish its general applicability. It demonstrated that the AYERA-Cas12a assay detects EHP with sensitivity surpassing that of conventional qPCR. Furthermore, the assay was engineered into a convenient one-pot format, significantly enhancing its practicality for field deployment. By offering a rapid, accurate, and easily implementable diagnostic tool, this research supports proactive environmental monitoring and hazard management, enabling timely interventions to mitigate economic losses, limit pathogen dissemination, and reduce the environmental impact of aquaculture.

## 2. Materials and Methods

### 2.1. Materials and Reagents

The ERA isothermal amplification kit, Cas12a protein, and 10× reaction buffer was purchased from XianDa Gene Technology (Suzhou, China). Quantitative polymerase chain reaction (qPCR) was performed using a fluorescence qPCR system (Zhenghegu Biotechnology, Ningbo, China). DNA samples from *Litopenaeus vannamei* were extracted using the Magen Universal DNA Extraction Kit (Magen Biotech Co., Ltd., Guangzhou, China). Fluorescence signals were monitored with a Rotor-Gene 6000 qPCR instrument (Corbett Research, Sydney, Australia), and imaging was performed using a 470 nm LED transilluminator (Labshark, Changde, China). Agarose gel electrophoresis was conducted using Gel Doc XR+ Gel Documentation System (Bio-Rad, Hercules, CA, USA). All pathogen-containing samples used in this study were laboratory-maintained isolates (Ningbo, China).

### 2.2. Design of ERA Primers, crRNAs, and Plasmid Templates

Two EHP-specific genes *ssu* and *swp*1 (GenBank accession No. MG015710.1, OR162445, and KX258197.1) were selected from the NCBI database. All six EHP genomes available in NCBI at the time of primer design were aligned. The primer-binding regions showed 100% sequence identity in all six genomes, with no SNPs or InDels. BLASTn searches against the shrimp genome and common aquatic pathogens revealed no significant homology. This ensures high specificity of the primers and crRNAs. ERA primer pairs were designed using Primer Premier 5 and validated for specificity via NCBI Primer-BLAST. Three crRNAs were designed: one PAM-restricted (TTTV) and two crRNA variants designed for PAM-independent ssDNA recognition, targeting the amplified regions. Plasmid templates were constructed based on the target amplicons (Table S1). All crRNAs, ssDNA-FQ reporters, primers, and probes were synthesized and purified by Shanghai Sangon Biological Engineering Technology and Services Co., Ltd. (Shanghai, China).

### 2.3. ERA Primer Screening

Standard ERA was performed according to the manufacturer’s instructions. Each reaction consisted of 20 µL of ERA resuspension buffer, 20 µL of nuclease-free water, and 2.5 µL each of forward and reverse primers (10 µM) (Supplementary Table S1). After thorough vortexing, the mixture was equally aliquoted into four PCR tubes, each pre-loaded with 0.75 µL of plasmid template (1 ng/µL) and 0.5 µL of activator in the cap. The tubes were briefly centrifuged and incubated at 40 °C for 14 min in a metal heating block. The amplification reaction was terminated by adding 1 µL of protease and incubating at 56 °C for 5 min. Amplification products were analyzed by electrophoresis on a 2% agarose gel. Primer pairs generating a single band of the expected size were selected for subsequent assays.

### 2.4. Optimization of Asymmetric ERA Primer Ratios

The concentration of the reverse primer was fixed at 10 µM, while the forward primer was titrated across a range of concentrations (500, 250, 166.7, 125, 100, and 80 nM), resulting in forward-to-reverse primer ratios of 1:20, 1:40, 1:60, 1:80, 1:100, and 1:120, respectively. Isothermal amplification was performed using the ERA system and reaction conditions described in Section 2.3. The amplification efficiency for each condition was evaluated by gel electrophoresis.

### 2.5. Validation of ssDNA for Cas12a Activation

To validate the feasibility of activating Cas12a with single-stranded DNA (ssDNA) generated via asymmetric ERA (aERA), a partitioned-reaction format was employed. The PAM-independent activation in our system does not rely on engineered Cas12a variants. Instead, it exploits the intrinsic ability of the Cas12a-crRNA complex to recognize and bind to ssDNA targets without requiring a PAM sequence [35]. To achieve this, we designed two PAM-independent crRNAs (AR-crRNA1 and AR-crRNA2) that are fully complementary to the expected ssDNA amplicons, and compared them against one PAM-independent crRNA (crRNA1) (Supplementary Table S1). For the PAM-independent crRNA, isothermal amplification was performed using the standard ERA system (Section 2.3). For the PAM-independent crRNAs, aERA was conducted to produce ssDNA amplicons, following the same core protocol but with a modified primer ratio: the forward and reverse primers were used at a final concentration ratio of 1:40 (0.25 µM forward: 10 µM reverse). After amplification and protease treatment, the products from both formats were incubated separately at 43 °C with the corresponding crRNA/Cas12a complex in a buffer containing a fluorescent reporter. The final concentrations in the detection mixture were 1 μM Cas12a, 1 μM crRNA (1:1 molar ratio), and 10 μM ssDNA-FQ reporter. Fluorescence kinetics were recorded every 60 s over 15 cycles using a Rotor-Gene 6000 real-time PCR system, and endpoint results were visualized under blue light transillumination. Thus, PAM independence is a property of the activation substrate (ssDNA) and crRNA design, not a modification of the Cas12a protein itself.

### 2.6. AYERA-Cas12a Multiplex Assay for Primer Ratio Analysis

The aERA amplification system and reaction conditions were established according to Section 2.3, with the forward/reverse primer ratio set as outline in Section 2.4. For the CRISPR/Cas12a detection step, AR-crRNA1 was employed as the guiding RNA. The composition of the detection system, incubation conditions, and signal measurement procedures adhered to the protocol outlined in Section 2.5.

### 2.7. AYERA-Cas12a System Optimization

The AYERA-Cas12a reaction was initially assembled by reconstituting the ERA dry powder with 20 µL of dissolution buffer and 10 µL of nuclease-free water, followed by the addition of 2.5 µL each of forward primer (0.25 µM) and reverse primer (10 µM), 30 µL of 1× Reaction Buffer, 3 µL of crRNA (1 µM), 3 µL of Cas12a protein (1 µM), 3 µL of ssDNA-FQ reporter (10 µM). The mixture was homogenized by gentle pipetting and equally aliquoted into four tubes. Subsequently, 0.75 µL of plasmid template (1 ng/µL) and 0.75 µL of activator were added to each tube and mixed thoroughly, with four replicates per sample. Optimized parameters included primer concentrations (250 nM, 500 nM, 1 µM, 5 µM, and 10 µM), Cas12a protein concentrations (250 nM, 500 nM, 1 µM, and 2 µM), Cas12a-to-crRNA molar ratios (4:1, 2:1, 1:1, and 1:2), and incubation temperatures (37, 40, 43, 46, 49, and 52 °C). Fluorescence kinetics were recorded every 60 s over 15 cycles using a Rotor-Gene 6000 real-time PCR system. Endpoint results were visualized under blue light transillumination and documented via smartphone imaging.

### 2.8. Feasibility of AYERA-Cas12a

To validate the one-pot AYERA-Cas12a system, aERA amplicons and symmetric ERA amplicons were combined with a PAM-independent Cas12a detection system in a single tube, using EHP DNA as the template. The reaction setup followed the protocol described in Section 2.6. Negative controls were prepared by omitting essential reaction components. Reactions were incubated at 46 °C for 9 min, and results were visualized under blue light.

### 2.9. Specificity and Sensitivity

The specificity of the AYERA-Cas12a system was evaluated using DNA extracted from shrimp infected with EHP, *Vibrio parahaemolyticus* (VP), White spot syndrome virus (WSSV), Infectious hypodermal and hematopoietic necrosis virus (IHHNV), Decapod iridescent virus 1 (DIV1), *V. parahaemolyticus* causing AHPND (*V*_AHPND_) and High pathogenic Vibrio (HLV), with nuclease-free water included as a blank control. For sensitivity assessment, the qPCR assay was tested using serial dilutions of the target plasmid at concentrations of 7 × 10^7^, 7 × 10^5^, 7 × 10^3^, 70, 35, 15, and 10 copies/µL. Similarly, the AYERA-Cas12a assay was assessed using plasmid concentrations of 10^5^, 10^4^, 10^3^, 10^2^, 30, 10^1^, and 10^0^ copies/µL. The sensitivity of both assays was compared. For accurate LoD determination, template concentrations near the expected LoD were tested with 20 technical replicates per concentration. The LoD was defined as the lowest concentration at which at least 95% of replicates produced a positive fluorescence signal. This criterion follows international guidelines for LoD validation in nucleic acid amplification tests.

### 2.10. Clinical Validation

qPCR-verified EHP-positive *L. vannamei* samples were incubated with the AYERA-Cas12a system at 46 °C for 6, 9, 12, or 15 min to determine the minimal detection time. The qPCR detection of EHP was performed using a commercial kit (Zhejiang Zhenghegu Biotechnology Co., Ltd., Ningbo, China; Cat. No. X-EHP-48). The amplification program was initial denaturation at 95 °C for 2 min, followed by 40 cycles of 95 °C for 5 s and 60 °C for 15 s. The assay was performed according to the manufacturer’s instructions. Subsequently, 56 clinical samples collected from multiple farms were tested under the optimized conditions, and the results were compared with those obtained by qPCR.

### 2.11. Statistical Analysis

All data were analyzed using GraphPad Prism 10 (GraphPad Software, San Diego, CA, USA). Statistical significance (*p* < 0.05) was determined by one-way ANOVA followed by Duncan’s multiple range test. Results from triplicate experiments are expressed as Mean ± Standard deviation.

## 3. Results

### 3.1. Design of the One-Pot AYERA-Cas12a Detection System

**Figure 1:**
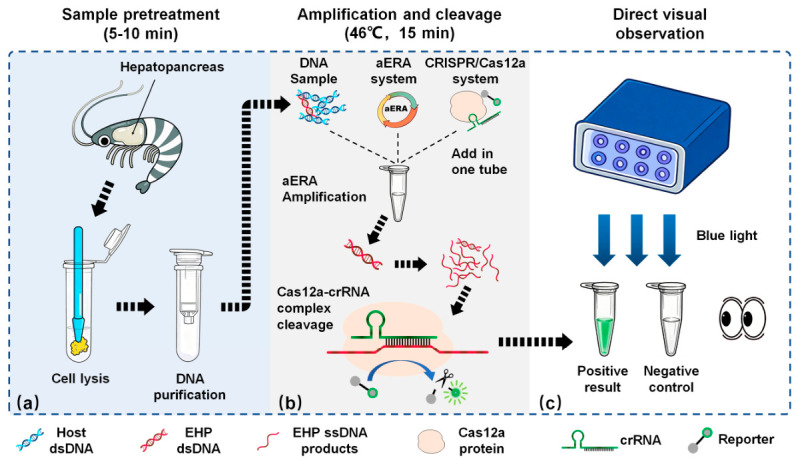
Detection Principle of the integrated AYERA-Cas12a system for nucleic acid detection. (**a**) The shrimp hepatopancreas homogenized with a pestle in a centrifuge tube releases DNA via mechanical lysis, purified by centrifugal column for subsequent detection applications within 5–10 min. (**b**) A single-tube co-amplification and cleavage assay integrating AYERA-Cas12a systems, incubated at 46 °C for 15 min. DNA samples are combined with both systems, where target DNA cleavage mediated by Cas12a-cRNA complexes generates a green fluorescent signal, enabling detection. (**c**) Signal transduction occurs via detection modules (e.g., optical readers), yielding a positive (+) or negative (−) outcome based on target presence.

This study established a PAM-unconstrained diagnostic paradigm by integrating aERA and CRISPR/Cas12a within a single reaction vessel. Target double-stranded DNA undergoes recombinase-facilitated strand invasion followed by single-stranded DNA binding protein (SSB)-stabilized primer extension, initiating ERA-mediated amplification. The subsequent depletion of limiting primers enables asymmetric amplification dominated by unrestricted primers, resulting in the generation of abundant single-stranded DNA (ssDNA) amplicons. The engineered PAM-independent crRNA complexed with Cas12a recognizes these ssDNA products, triggering dual nuclease activities, including sequence-directed cis-cleavage of target DNA and collateral trans-cleavage of bystander ssDNA-FQ reporters. Fluorophore liberation yields detectable fluorescence under UV transillumination (Figure 1), where positive specimens exhibit emission while negative controls maintain a quenched background. This strategic design eliminates Cas12a recognition of dsDNA intermediates, which inherently require PAM motifs, thereby preventing premature cleavage events that terminate amplification and fundamentally resolving the intrinsic incompatibility between isothermal nucleic acid amplification and CRISPR systems in single-tube configurations.

### 3.2. aERA Primer Design and Optimization

**Figure 2:**
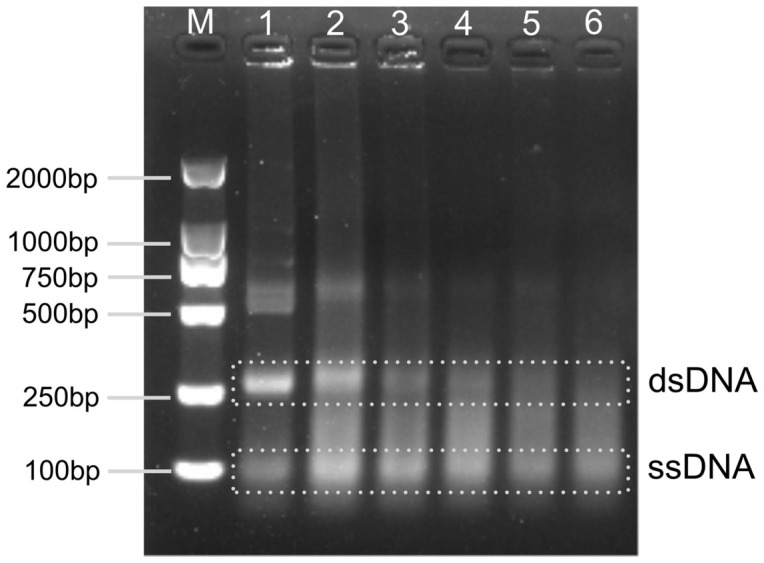
Visualization of aERA amplification on agarose gel. (1–6) Primer ratios ranging from 500 nM:10 μM (1:20) to 80 nM:10 μM (1:120). (M) DL2000 Ladder Marker.

Based on the EHP-3 primers selected from the ERA primer screening experiments (Figure S1), the concentration ratios of 1:20, 1:40, 1:60, 1:80, 1:100, and 1:120 were optimized. Gel electrophoretic analysis revealed distinct bimodal banding patterns, with upper and lower bands corresponding to double-stranded and single-stranded DNA products, respectively (Figure 2). Critical examination demonstrated a progressive intensification of ssDNA bands concomitant with dsDNA band attenuation at ratios of 1:20 and 1:40, establishing the limiting primer concentration as the primary determinant of ssDNA production efficiency. Beyond the 1:40 threshold, ssDNA bands progressively diminished while dsDNA amplification became undetectable. Consequently, the 1:40 ratio (10 μM limiting primer: 250 nM excess primer) was identified as optimal for subsequent experimental workflows, balancing maximal ssDNA yield with robust amplification kinetics.

### 3.3. Optimization of the AYERA-Cas12a Detection System

**Figure 3:**
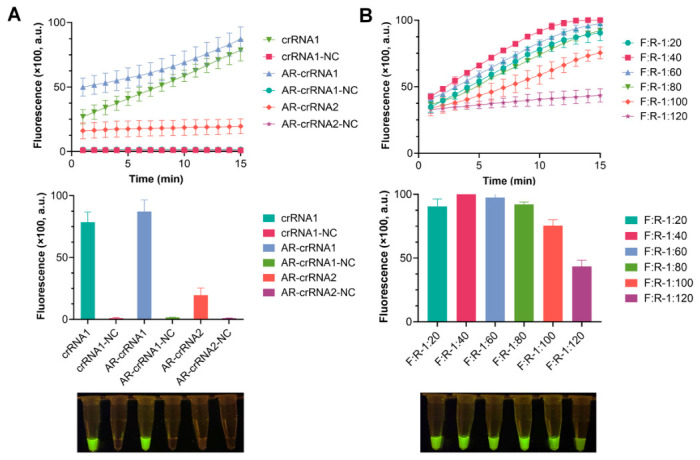
Feasibility of ssDNA activation of the Cas system and optimization of aERA primer ratios. (**A**) CRISPR/Cas12a cleavage efficiency comparison of three crRNA variants (AR-crRNA1, AR-crRNA2, crRNA1) with distinct PAM requirements (TTTA restriction) under optimized aERA amplification conditions. (**B**) Using the AYERA-Cas12a detection system, perform a multiplexed assay to measure the fluorescence signal values and fluorescence images for different ERA primer concentration ratios. Error bars represent the mean ± standard deviation of triplicate experiments.

**Figure 4:**
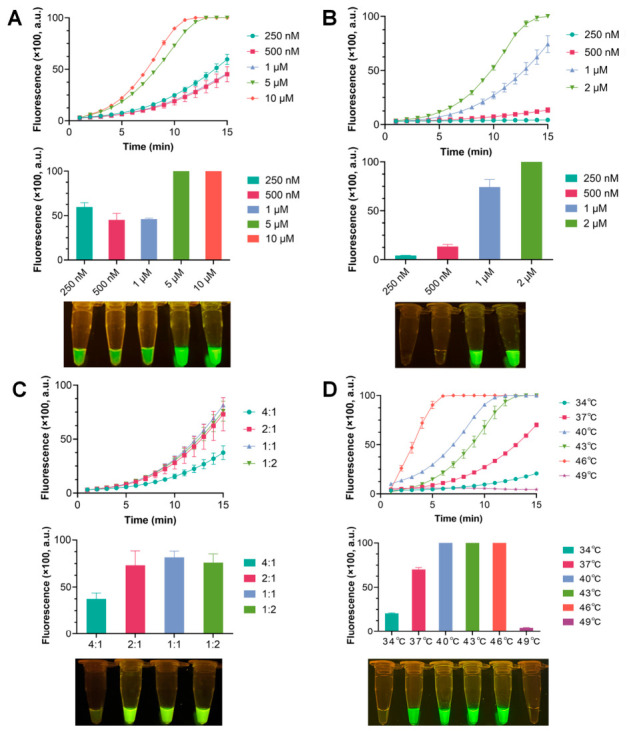
Optimization of AYERA-Cas12a reaction conditions. (**A**) Real-time curves, bar charts, and fluorescence images for primer concentration optimization. (**B**) Real-time curves, bar charts, and fluorescence images for Cas12a protein concentration optimization. (**C**) Real-time curves, bar charts, and fluorescence images for Cas12a protein-to-crRNA concentration ratio optimization. (**D**) Real-time curves, bar charts, and fluorescence images for reaction temperature optimization. Error bars represent the mean ± standard deviation of triplicate experiments.

Fluorescence analysis demonstrated that AR-crRNA1 not only triggered a detectable signal but also exhibited enhanced kinetics and intensity compared to crRNA1, confirming ssDNA-mediated Cas activation via PAM-independent recognition (Figure 3A). Subsequent optimization using AR-crRNA1 revealed that primer concentration ratios directly influenced detection performance: the 1:40 ratio achieved maximal fluorescence amplitude and initial velocity, with progressive signal attenuation observed at higher ratios (1:60–1:120) (Figure 3B). Gel electrophoretic correlation confirmed that 1:40 is optimal for ssDNA yield (Figure 2). Further systematic refinement of the integrated one-pot system identified 5 μM primer concentration as providing peak signal intensity at 13 min (Figure 4A), while balancing reagent economy. A protein concentration of 1 μM Cas12a delivered optimal signal-to-background fluorescence (Figure 4B). And a 1:1 Cas12a: crRNA molar ratio achieved rapid kinetic onset despite equivalent endpoint signals across tested ratios (Figure 4C). Under the optimized conditions (5 μM primers, 1 μM Cas12a, 1:1 Cas12a: crRNA molar ratio, 46 °C), maximum detection signal was achieved within 6 min (Figure 4D).

### 3.4. Validation of AYERA-Cas12a System Feasibility

**Figure 5:**
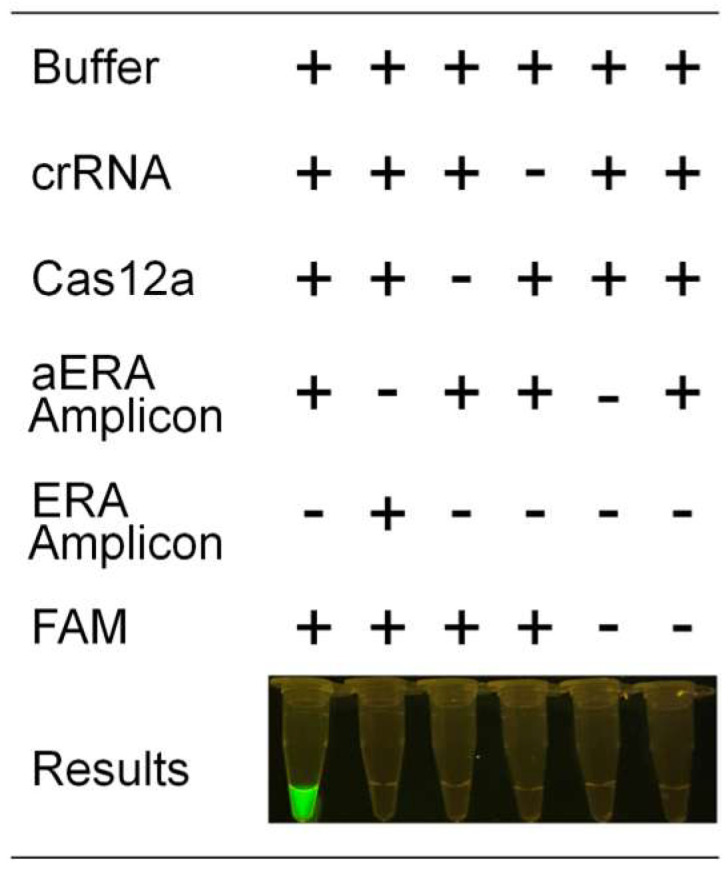
Feasibility of AYERA-Cas12a integrated fluorescence validation.

Post-amplification incubation (9 min under isothermal conditions) followed by blue light transillumination analysis revealed exclusive fluorescence emission in the configuration that combined aERA amplicons with the fully reconstituted PAM-independent Cas12a complex, in contrast to four fluorescence-deficient control assemblies (Figure 5). Notably, conventional ERA products exhibited complete resistance to cleavage by the PAM-independent crRNA-Cas12a machinery. These observations collectively validate the operational feasibility of the integrated AYERA-Cas12a platform, confirming its dependency on both asymmetric amplification products and functional Cas12a-mediated collateral activity for diagnostic signal generation.

### 3.5. Analytical Specificity and Detection Sensitivity

**Figure 6:**
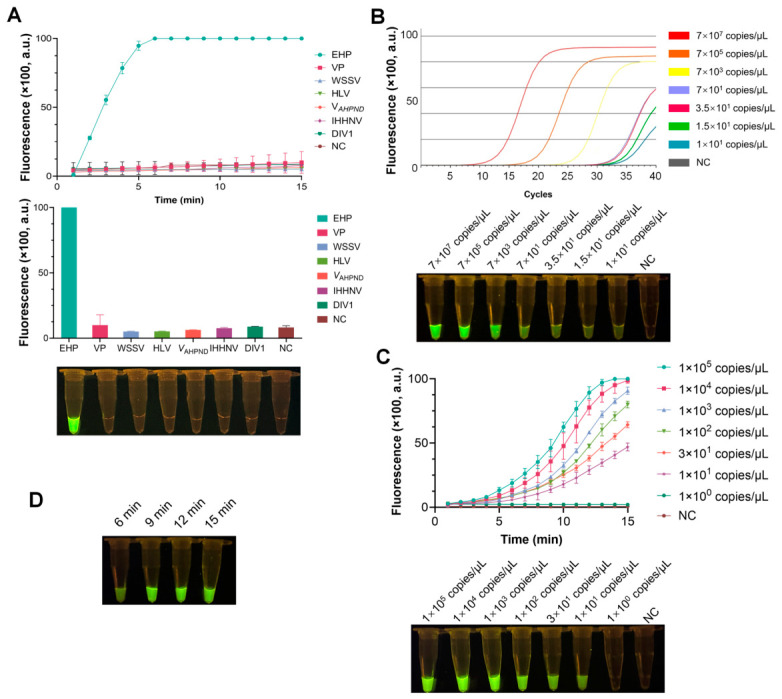
Specificity, sensitivity, and detection time of AYERA-Cas12a. (**A**) AYERA-Cas12a specificity evaluation: real-time curve, bar chart, and fluorescence image. (**B**) qPCR sensitivity evaluation: real-time curve and fluorescence image. (**C**) Real-time curve and fluorescence image for sensitivity evaluation of AYERA-Cas12a. (**D**) Fluorescence images of actual samples were detected between 6 and 9 min. Error bars represent the mean ± standard deviation of triplicate experiments.

The diagnostic specificity of the AYERA-Cas12a platform was rigorously evaluated through a comprehensive cross-reactivity assessment using DNA extracted from *L. vannamei* infected with EHP, VP, WSSV, IHHNV, DIV1, *V*_AHPND_ and HLV. Distinctive fluorescence emission was exclusively observed in EHP-positive reactions under 470 nm wavelength transillumination (Figure 6A), validating the exceptional target specificity, with no fluorescence signal detected from other non-target pathogens. The results confirm that the AYERA-Cas12a assay demonstrates high specificity in detecting EHP. Sensitivity assessment using serially diluted plasmid standards established a consistent limit of detection (LOD) of 10 copies/μL for both the AYERA-Cas12a assay and qPCR (Figure 6B,C). At 10 copies/μL, 19 out of 20 replicates (95%) were positive, confirming the LoD as 10 copies/μL. At 30 copies/μL, all 20 replicates (100%) were positive. No positive signals were detected at 1 copy/μL (10^0^ copies/μL). The integrated CRISPR platform achieved this LOD within 15 min, whereas qPCR required 45 min, representing a threefold reduction in detection time.

### 3.6. Clinical Validation and Field Deployment

**Figure 7:**
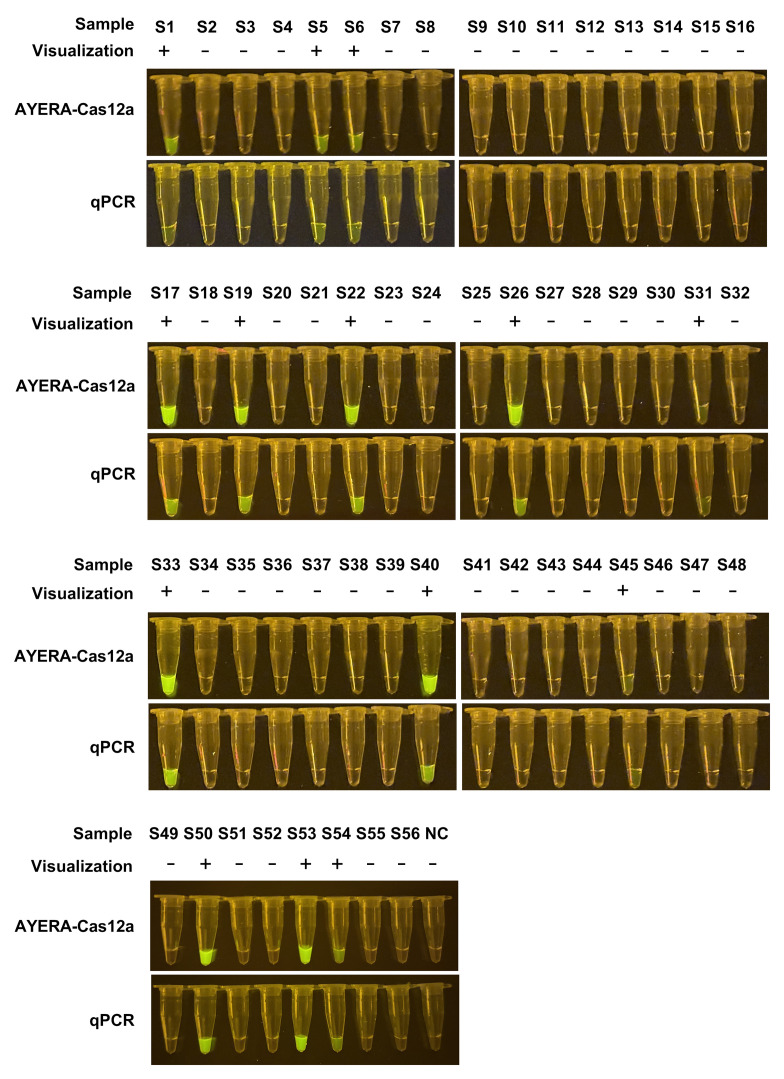
Practical Application of AYERA-Cas12a. Fluorescence images of AYERA-Cas12a and qPCR detection for 56 shrimp samples and 1 Negative control.

The detection kinetics were initially assessed using one qPCR-validated clinical specimen from infected *L. vannamei* under isothermal incubation at 46 °C. Fluorescence intensity measurements at 6, 9, 12, and 15 min indicated initial signal emergence at 6 min, peaking at 9 min (Figure 6D), thus the detection time was set at 6 min for subsequent large-scale clinical sample testing. Subsequently, A total of fifty-six clinical samples (14 EHP-positive and 42 EHP-negative, as confirmed by qPCR) were collected from three different farms in Ningbo and re-tested using the AYERA-Cas12a assay alongside a non-template control. The results showed 100% diagnostic agreement with qPCR (Figure 7), validating the assay’s operational robustness for field surveillance.

## 4. Discussion

The CRISPR-Cas12a detection system employs Cas12a protein and crRNA to specifically recognize target DNA. Upon activation, Cas12a cleaves single-stranded DNA probes, generating detectable fluorescent or visual signals [36]. However, the sensitivity of CRISPR-Cas12a alone is often inadequate for low-concentration targets, limiting its application in point-of-care settings. Isothermal nucleic acid amplification techniques, such as RPA, can rapidly amplify targets at a constant temperature without requiring specialized equipment, but they may produce false-positive results due to nonspecific amplification [37]. The integration of isothermal amplification with CRISPR-Cas12a combines the sensitivity of pre-amplification with the high specificity of CRISPR-mediated recognition. This combined approach significantly enhances detection sensitivity, reduces false positives, and expands the utility of CRISPR-based diagnostics in resource-limited environments. This study developed a rapid, sensitive, and visual detection method for EHP based on the aERA CRISPR/Cas12a system. The method significantly reduces detection time, simplifies operations, and eliminates the need for complex instrumentation.

The primary challenge in constructing a one-pot Cas12a system lies in overcoming its limited reaction compatibility. This incompatibility arises because the Cas12a system can be activated during the early stages of isothermal amplification, leading to specific cleavage of target sequences containing PAM motifs. This premature cleavage depletes the amplification template, thereby inhibiting the nucleic acid amplification process [38]. Studies have confirmed that the Cas12a system can recognize ssDNA target sites without a PAM requirement, activating both specific and nonspecific cleavage activities [39,40]. This principle allows for assay design in which a small amount of initial target is amplified into a large quantity of ssDNA via asymmetric amplification. The Cas12a system then shifts its recognition from PAM-containing double-stranded targets to these ssDNA products. The resulting specific cleavage does not interfere with the initial phase of the isothermal amplification. The effective coupling with the Cas system is highly dependent on the concentration of ssDNA present. The production of ssDNA relies on aERA, a method that employs a pair of primers at unequal concentrations to generate abundant ssDNA [41]. Consequently, the concentration ratio of the forward and reverse primers is critical for efficient ssDNA yield. Our results identified an optimal primer concentration ratio of 1:40 (10 µM:250 nM). While prior research suggested that a ratio of 1:320 could produce more ssDNA, our experimental data indicated that an excessively high ratio adversely affected the initial amplification of dsDNA [42]. It was hypothesized that as the primer concentration ratio in the asymmetric amplification increases, ssDNA yield initially rises and then declines, whereas dsDNA production remains relatively stable. The underlying mechanism is that efficient ssDNA synthesis depends on the prior accumulation of a sufficient amount of dsDNA template. When the primer ratio exceeds a critical threshold, the efficiency of initial dsDNA amplification may be compromised, subsequently limiting ssDNA production. In this study, an aERA-based amplification method for EHP was established. By optimizing the efficiency of ssDNA production, this method enables the subsequent CRISPR/Cas12a detection system to exhibit its trans-cleavage activity more rapidly and at an earlier stage.

To fundamentally address the incompatibility between amplification and detection in one-pot Cas12a assays caused by PAM dependency, alternative PAM-free strategies have been developed to decouple Cas12a activation from endogenous genomic PAM sequences. One approach involves engineering Cas12a variants or mining metagenomic data to identify natural PAM-less orthologs, such as the reconstructed common ancestor ReChb or the high-activity plant editor Mb2Cas12a, which provides a fundamental solution [43,44]. However, the extensive characterization, system re-optimization, and stability assessment necessary for these novel proteins currently render them unsuitable for routine application. Alternatively, the design of specially structured DNA activators, such as PAM-independent hairpin structures that trigger Cas12a’s trans-cleavage, provides a novel pathway for high-specificity sensing, although it requires custom probe synthesis for each target [35,45]. Additionally, enzymatic processing strategies, for instance, using exonucleases to generate activatable DNA substrates, present clever solutions but introduce additional enzymatic components, increasing the complexity of one-pot reactions [45]. Consequently, the fourth strategy, which involves directly controlling amplicon type via asymmetric amplification, stands out for its integrated advantages [46]. Our approach directly addresses the core incompatibility described above by combining asymmetric ERA (aERA) with a PAM-independent crRNA. In symmetric ERA, both primers are used at equal concentrations, generating dsDNA as the primary product. When such dsDNA is present, Cas12a activation would require a PAM motif on the target, and any early activation would cleave the template. By contrast, aERA uses a limiting forward primer (250 nM) and an excess reverse primer (10 μM), producing a large amount of ssDNA after the initial dsDNA accumulation. Critically, the Cas12a-crRNA complex we designed (AR-crRNA1) recognizes this ssDNA directly without any PAM requirement [47]. As demonstrated in Figure 5, symmetric ERA products failed to activate the PAM-independent Cas12a complex, whereas abundant ssDNA from aERA triggered a strong fluorescent signal. Thus, our system decouples Cas12a activation from the dsDNA intermediates, preventing premature template cleavage and allowing simultaneous amplification and detection in a single tube. This mechanistic design differs fundamentally from physical isolation or enzyme engineering strategies: we do not separate the reactions, nor do we mutate the Cas protein. Instead, we redirect the activation pathway from dsDNA-dependent, PAM-restricted recognition to ssDNA-mediated, PAM-free activation, achieving intrinsic compatibility with minimal additional complexity. The results demonstrated that AR-crRNA1 not only effectively activated the trans-cleavage activity of Cas12a but also exhibited enhanced activation kinetics and signal intensity compared to traditional PAM-dependent crRNA (Figure 3A). This confirms that rational crRNA design can completely bypass the constraints imposed by endogenous PAM sequences, enabling direct activation of Cas12a by ssDNA amplicons. Decoupling this mechanism is crucial, as it frees the detection system from reliance on specific PAM sequences within the target DNA, thereby significantly broadening the detectable target range and application scenarios for the CRISPR-Cas12a system. This provides compelling evidence for the necessity of the synergistic interplay between asymmetric amplification and PAM-independent crRNA, which together form the foundation for the high sensitivity and specificity of this platform.

In terms of performance comparison, the AYERA-Cas12a platform demonstrates a balance between speed and sensitivity. It achieves a sensitivity of 10 copies per microliter, with a typical detection time of 15 min. Notably, for actual sample testing, the platform can deliver results in as little as 6 min (Figure 7). Compared to other mainstream one-pot CRISPR detection technologies, it holds a significant advantage in detection speed (Table 1). For instance, systems employing physical isolation strategies, such as the Glycerol or DAMR systems, require up to 60 min, while the OAR-CRISPR platform, also based on asymmetric RPA, offers comparable speed at 24 min but with lower sensitivity than our platform. Although technologies like HPL-opCRISPR (0.2 copies/μL) or TOP-CRISPR (0.5 copies/μL) achieve higher absolute sensitivity, this typically comes at the cost of more complex system designs, such as dual-lyophilized microspheres or primer-generated crRNA (Table 1). The platform addresses the compatibility issue through “substrate engineering” rather than “physical isolation”, with abundant ssDNA generated directly via asymmetric ERA enabling efficient activation of the PAM-independent Cas12a/crRNA complex. This mechanism not only circumvents the amplification inhibition by Cas proteins inherent in traditional methods but also leverages faster hybridization kinetics between ssDNA and crRNA. Consequently, it accelerates the detection cascade, ultimately achieving a breakthrough in reducing detection time to the minute scale while maintaining high sensitivity. A detection limit of 10 copies/μL is clinically relevant for routine EHP surveillance because infected shrimp typically harbor much higher parasite loads, and the practical need for sub-copy/μL sensitivity remains unclear.

Nevertheless, this study has certain limitations and avenues for future development. Firstly, although a sensitivity of 10 copies/μL meets the requirements for most rapid on-site screenings, there is scope for improvement for monitoring very early-stage EHP infections compared to the most sensitive technologies. We acknowledge that precise LoD determination ideally requires a larger number of replicates, especially for stochastic low-copy amplification. Our expanded analysis using 20 replicates at near-LoD concentrations provides a more robust estimate than the initial assessment, yet further validation with additional replicates across multiple runs would further strengthen confidence in the LoD. Future work could focus on optimizing lyophilization protocols to enhance reagent stability in aquaculture farm environments, exploring more efficient reporter systems, or incorporating additional signal amplification strategies to further lower the limit of detection. Secondly, the current validation is confined to purified DNA samples from a single pathogen (EHP). Its anti-interference capability in actual complex aquaculture matrices (e.g., shrimp hepatopancreas, feces, or pond water) and its potential for multiplex synchronous detection alongside other critical shrimp pathogens such as WSSV and DIV1, require further systematic evaluation. This is crucial for the rapid differential diagnosis of co-infections in farm settings. Furthermore, to achieve a true “sample-to-answer” field application, the integration of rapid nucleic acid extraction steps suitable for shrimp tissue or water samples with this detection system, or the development of simpler sample lysis and release methods, represents a key challenge for the next phase, aiming to reduce reliance on specialized equipment and procedures. Finally, the technical framework of this platform has potential for broader application, but for each new target, the primers and crRNA must be re-optimized and the system re-validated. Expanding this paradigm to different CRISPR-Cas systems (e.g., Cas13, Cas14) would require systematic evaluation and is not guaranteed to be directly transferable.

## 5. Conclusions

In summary, this study successfully developed the AYERA-Cas12a platform by integrating asymmetric ERA (aERA) with a PAM-independent CRISPR/Cas12a system. The key mechanistic insight is that aERA generates abundant single-stranded DNA (ssDNA) amplicons, which serve as the direct activation substrate for Cas12a without requiring any PAM sequence. This design decouples Cas12a activation from the initial double-stranded DNA templates, thereby preventing premature template cleavage and enabling genuine single-tube compatibility. The assay achieves ultra-rapid visual detection within 15 min with a limit of detection of 10 copies/µL, and shows high specificity for EHP against six other major shrimp pathogens. Validation with 56 clinical *L. vannamei* samples demonstrated 100% agreement with qPCR. With its simple procedure, isothermal conditions, and clear visual readout under blue light, the AYERA-Cas12a platform provides a reliable, rapid, and user-friendly tool for on-site surveillance and early diagnosis of EHP in shrimp aquaculture. This study illustrates how asymmetric amplification can resolve the inherent incompatibility between isothermal nucleic acid amplification and CRISPR detection, offering a generalizable strategy for one-pot molecular diagnostics.

## Figures and Tables

**Table 1:** Comparison of detection design, sensitivity, and speed among several CRISPR/Cas12a one-pot detection methods combined with isothermal amplification technology.

Name	Description	Sensitivity	Duration	Quote
Cas12aVDet	In a single tube, the Cas12a system was first immobilized on the wall of the tube, and the RPA system was reacted at the bottom of the tube for 15 min before centrifugation and mixing.	10 αM	30 min	[48]
Glycerol/RPA-CRISPR/Cas12a	The glycerol system was utilized to isolate the Cas12a system from the RPA system.	10 copies/μL	60 min	[30]
DAMR/RPA-CRISPR/Cas12a	The Cas12a system and the RPA system were combined in a single tube using sucrose concentration gradients.	10–100 copies/μL	60 min	[49]
HPL-opCRISPR	A dual lyophilized microsphere system was used to combine the Cas system and the RPA system.	0.2 copies/μL	30 min	[50]
Light control RPA-CRISPR/Cas12a	Control of crRNA using P-RNA binding to crRNA. Then release of crRNA by UV blockade of binding.	30 copies/μL	60 min	[51]
ERA-CRISPR/Cas12a	Introduction of the ERA process and optimization of reaction components.	10 copies/μL	40 min	[52]
Se-RPA-CRISPR	RPA was enhanced by the addition of selenium-modified adenosine triphosphate, which reduced the low compatibility of the Cas system with RPA.	169 αM	20 min	[53]
TOP-CRISPR	Incorporation of the crRNA template region and promoter template region in the RPA forward primer self-produces crRNA to achieve non-direct incorporation of crRNA and PAM dependence.	0.5 copies/μL	30 min	[54]
OPERA-Cas12a	The DSD statistical method was utilized to analyze the various factors affecting the one-tube reaction, and an optimization system applicable to different target sequences was proposed.	6 copies/μL	60 min	[38]
OAR-CRISPR	An asymmetric RPA process was introduced to combine with Cas12b.	60 copies/μL	24 min	[47]
AYERA-Cas12a	Introduction of an asymmetric ERA combined with Cas12a and optimization of reaction component concentrations.	10 copies/μL	15 min	this study

## Data Availability

The original contributions presented in this study are included in the article/Supplementary Materials. Further inquiries can be directed to the corresponding authors.

## Supplementary Materials

The following supporting information can be downloaded at: https://www.mdpi.com/article/10.3390/microorganisms14061307/s1, Figure S1: Specific ERA Primer Screening. This study conducted a comprehensive evaluation of four primer combinations to identify the optimal ERA primer pair. Using EHP-infected shrimp DNA at a concentration of 21 ng/μL as template, all primer combinations successfully amplified products of the expected size. Notably, the EHP-3 primer pair generated a 258 bp amplification fragment (S1), exhibiting a clear and distinct band. Based on the above results, we selected the EHP-3 primers for subsequent experiments. Table S1: Sequence list of primers, crRNA, single-stranded DNA targets, and FQ-reporter gene used in this study.

## Abbreviations

The following abbreviations are used in this manuscript:

aERA	Asymmetric enzymatic recombinase amplification
DIV1	Decapod iridescent virus 1
EHP	*Enterocytozoon hepatopenaei*
ERA	Enzymatic recombinase amplification
HLV	High pathogenic vibrio
IHHNV	Infectious hypodermal and hematopoietic necrosis virus
LFAs	Lateral flow assays
LOD	Limit of detection
LAMP	Loop-mediated isothermal amplification
POC	Point-of-care
POCT	Point-of-care testing
PCR	Polymerase chain reaction
qPCR	Quantitative PCR
RPA	Recombinase polymerase amplification
ssDNA	Single-stranded DNA
*V*_AHPND_	*V. parahaemolyticus* causing AHPND
VP	*Vibrio parahaemolyticus*
WSSV	White spot syndrome virus
