# Supplementary material for: Development of a Single-Tube Asymmetric ERA-CRISPR/Cas12a Assay for Rapid Visual Detection of Enterocytozoon hepatopenaei in Shrimp
Source: Microorganisms. 2026 Jun 11;14(6):1307. doi: 10.3390/microorganisms14061307 (PMC13303178; doi:10.3390/microorganisms14061307)
Supplement: Supplementary file 1 [file microorganisms-14-01307-s001.zip › microorganisms-4335422-supplementary.pdf]

Supplementary Information

Development of a Single-Tube Asymmetric ERA-CRISPR/Cas12a Assay for Rapid Visual Detection of *Enterocytozoon hepatopenaei* in Shrimp

Ren Liu [a], Sizhi Sun [b], Zhenyang Ma [a], Xin Zhou [a], Jiaojiao Han [a,*], Jun Zhou [a,*]

[a] State Key Laboratory for Quality and Safety of Agro-products, School of Marine Sciences, Ningbo University, Ningbo 315211, China

[b] Yinzhou Haohai Aquaculture Farm, Ningbo 315141, China

* Corresponding author

Dr. Jiaojiao Han

School of Marine Sciences, Ningbo University, 169 Qixing South Road, Ningbo, China

E-mail address: hanjiaojiao@nbu.edu.cn

Prof. Jun Zhou

School of Marine Sciences, Ningbo University, 169 Qixing South Road, Ningbo, China

E-mail address: zhoujun1@nbu.edu.cn

1. Supplementary Results

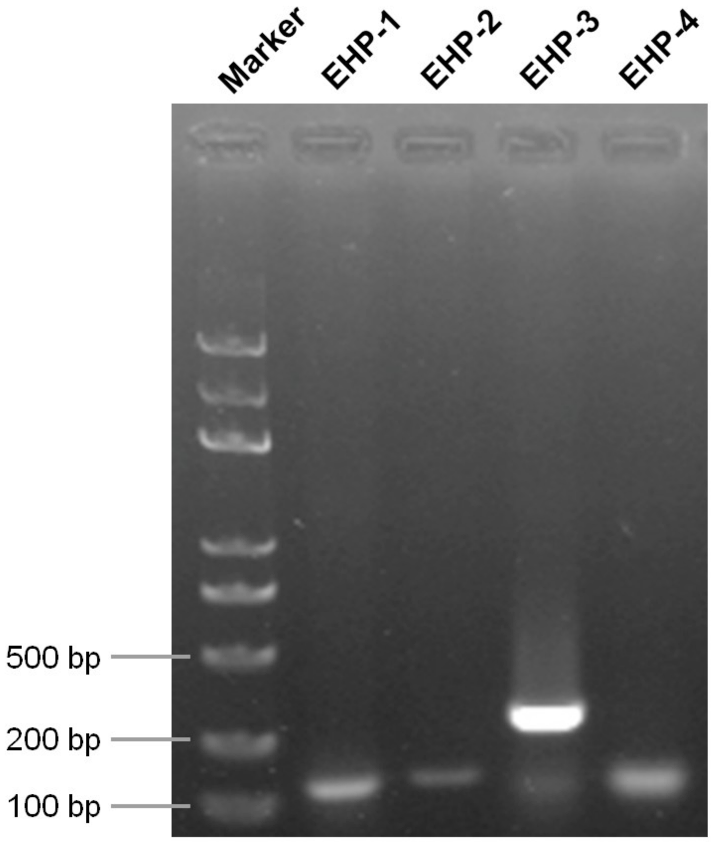

Figure S1. **Specific ERA Primer Screening.**

This study conducted a comprehensive evaluation of four primer combinations to identify the optimal ERA primer pair. Using EHP-infected shrimp DNA at a concentration of 21 ng/μL as template, all primer combinations successfully amplified products of the expected size. Notably, the EHP-3 primer pair generated a 258-bp amplification fragment (S1), exhibiting a clear and distinct band. Based on the above results, we selected the EHP-3 primers for subsequent experiments.

Electrophoresis analysis of ERA amplification products using ERA-specific primers from EHP. Electrophoresis results on a 2% agarose gel. Bands were visualized using the Bio-Rad XR+ imaging system.

Supplementary Tables

Table S1. Sequence list of primers, crRNA, single-stranded DNA targets, and FQ-reporter gene used in this study.

Item	Oligo name	Sequence (5'-3')
ERA primer	EHP-1 (*swp*1 gene)	5'-TGTTTTGCAGAGTGTTGTTAAGGGTTTA-3'<br>5'-ATCTTAGGTTATTTACAGTTTTGCGTTG-3'
	EHP-2 (*swp*1 gene)	5'-TATGAGCTTTCAAATACAGTTGGAGACA-3'<br>5'-ATCTTAGGTTATTTACAGTTTTGCGTTG-3'
	EHP-3 (*ssu* gene)	5'-CCTTTGAATAGAAGGAAGTGATACGATCC-3'<br>5'-CCTTCGACTCCTTCTCAATATGACTTTC-3'
	EHP-4 (*swp*1 gene)	5'-GTAGGATATGAGCTTTCAAATACAGTTGGAGAC-3'<br>5'-TTTTTTCTAAATTTTCTTTTTGATCTTCTT-3'
Asymmetric crRNA	ARcrRNA-1	UAAUUUCUACUAAGUGUAGAUAUCCGCCGUAUAC AGUAUGUAG
	ARcrRNA-2	UAAUUUCUACUAAGUGUAGAUGUGGCGACACUAA GCACGAA
Symmetrical crRNA	crRNA-1	UAAUUUCUACUAAGUGUAGAUUGAUAGCUAUAUA UGAAGGA
Plasmid DNA	OR162445	CCTTTGAATAGAAGGAAGTGATACGATCCTATTGTCG CAAGTTAGTTTATTTGTAGCAATATAGATAGATGAATA GGCAAGGAAAAGGGAAGAATTTAAATATTAATTCGA AGCTGCCCGAAGTGGCGACACTAAGCACGAATCCGC CGTATACAGTATGTAGTTATTGTATTTATTTATGATAGC TATATATGAAGGAGTGTAAATTTGCTCAAGGTATGGA GGCTCTGGAAAGTCATATTGAGAAGGAGTCGAAGG
FQ probe	ssDNA probe	(FAM) 5' -TTATT -3' (BHQ1)
